# Experimental pneumococcal carriage in people living with HIV in Malawi: the first controlled human infection model in a key at-risk population

**DOI:** 10.12688/wellcomeopenres.19949.1

**Published:** 2024-01-03

**Authors:** Klara Doherty, Dingase Dula, Anthony Chirwa, Edna Nsomba, Vitumbiko S. Nkhoma, Neema Toto, Tarsizio Chikaonda, Raphael Kamng'ona, Joseph Phiri, Jesús Reiné, John Ndaferankhande, Lumbani Makhaza, Peter Banda, Kondwani Jambo, Daniela M Ferreira, Stephen B Gordon

**Affiliations:** 1 Malawi-Liverpool-Wellcome Trust Clinical Research Programme, Blantyre, Southern Region, Malawi; 2 Liverpool School of Tropical Medicine, Liverpool, L3 5QA, UK; 3 Oxford Vaccine Group, University of Oxford, Oxford, England, UK; 4 Kamuzu University of Health Sciences, Blantyre, Malawi

**Keywords:** pneumococcal disease, people living with HIV, pneumococcal carriage, controlled human infection models, experimental pneumococcal carriage model, immune correlates of protection

## Abstract

**Background:** As well as suffering a high burden of pneumococcal disease people living with HIV (PLHIV) may contribute to community transmission in sub-Saharan African (sSA) settings. Pneumococcal vaccination is not currently offered to PLHIV in sSA but may prevent disease and reduce transmission. More evidence of vaccine effectiveness against carriage in PLHIV is needed. An Experimental Human Pneumococcal Carriage model (EHPC) has been safely and acceptably used in healthy adults in Malawi to evaluate pneumococcal vaccines against carriage and to identify immune correlates of protection from carriage. This study will establish the same model in PLHIV and will be the first controlled human infection model (CHIM) in this key population.

**Methods:** Healthy participants with and without HIV will be inoculated intranasally with *Streptococcus pneumoniae* serotype 6B. Sequential cohorts will be challenged with increasing doses to determine the optimal safe challenge dose to establish experimental carriage. Nasal fluid, nasal mucosal, and blood samples will be taken before inoculation and on days 2, 7, 14, and 21 following inoculation to measure pneumococcal carriage density and identify immune correlates of protection from carriage. The vast majority of natural pneumococcal carriage events in PLHIV do not result in invasive disease and no invasive disease is expected in this study. However, robust participant safety monitoring is designed to identify signs of invasive disease early should they develop, and to implement treatment immediately. Participants will complete a Likert-style questionnaire at study-end to establish acceptability.

**Interpretations:** We expect the EHPC model to be safely and acceptably implemented in PLHIV. The CHIM can then be used to accelerate pneumococcal vaccine evaluations in this population, and an evidence-based pneumococcal vaccination policy for PLHIV in sSA.

## Introduction

Controlled human infection models (CHIMs) involve the introduction of an infective agent to healthy volunteer participants in order to observe disease pathophysiology and evaluate vaccine efficacy. CHIMs in sub-Saharan Africa have demonstrated safety, feasibility, and acceptability, and have accelerated vaccine evaluations and development for pneumococcus and malaria ^1, 2^. Vaccine studies using CHIMs are cheaper and quicker than large clinical trials which rely on community exposure to pathogens in order to test vaccines, making them a promising tool in resource-limited settings or when disease incidence is very low ^3^. To date CHIMs have largely been implemented in young, healthy adults; the population with generally the lowest burden of infectious disease. It is important that CHIM studies be expanded to populations most affected by the disease, provided that this can be done safely. This will be the first study to implement a CHIM in a key at-risk population in sub-Saharan Africa, and for a disease that continues to represent a large burden of disease worldwide ^4^.

People living with HIV (PLHIV) are one of the most important populations to consider when addressing the burden of pneumococcal disease. They have a high burden of pneumococcal nasopharyngeal carriage, particularly if established on antiretroviral therapy (ART), with 26 to 52% of PLHIV in sub-Saharan Africa exhibiting pneumococcal carriage at any one time ^5, 6^. Pneumococcal nasopharyngeal carriage is a prerequisite for invasive pneumococcal disease such as pneumonia and septicaemia, and is required for onward transmission of the bacteria in the community ^7^. The high burden of pneumococcal carriage in PLHIV may form a reservoir for transmission in sub-Saharan Africa, and may be driving the ongoing pneumococcal carriage observed in vaccinated populations ^8– 11^. There is currently no pneumococcal vaccine policy for addressing pneumococcal carriage nor pneumococcal disease in PLHIV in sub-Saharan Africa. The 23-valent pneumococcal polysaccharide vaccine (PPV23) is associated with harm in PLHIV ^12, 13^, and while the 7-valent pneumococcal polysaccharide-conjugate vaccine (PCV7) is safe and effective for preventing vaccine-serotype disease ^14^, it has limited serotype-coverage ^15^. More evidence is needed to guide an evidence-based pneumococcal vaccination policy for PLHIV. The experimental human pneumococcal carriage (EHPC) model was established at the Malawi-Liverpool Wellcome Trust programme (MLW) in 2019 in order to accelerate vaccine evaluations and development. It has demonstrated acceptability to local stakeholders, and has successfully been used to evaluate vaccine efficacy for preventing pneumococcal carriage in healthy adults ^15^. This study will establish the EHPC in PLHIV with the translational aim of facilitating pneumococcal vaccine studies in this key population.

## Study protocol

### Study objectives

1. Establish the safety of experimental pneumococcal inoculation in PLHIV and the acceptability of the procedure to participants.
2. Establish the rate and density of pneumococcal carriage in PLHIV compared to HIV negative adults after experimental pneumococcal inoculation.
3. Identify the immune correlates of protection from pneumococcal carriage in PLHIV following experimental pneumococcal inoculation.

### Study hypotheses

- PLHIV will exhibit higher carriage rate and density than HIV-negative controls following experimental pneumococcal inoculation.
- PLHIV will have perturbed mucosal and systemic immunity in response to experimental pneumococcal inoculation, compared to HIV-negative controls, which will predispose them to pneumococcal carriage.

### Study design

**Figure 1.**
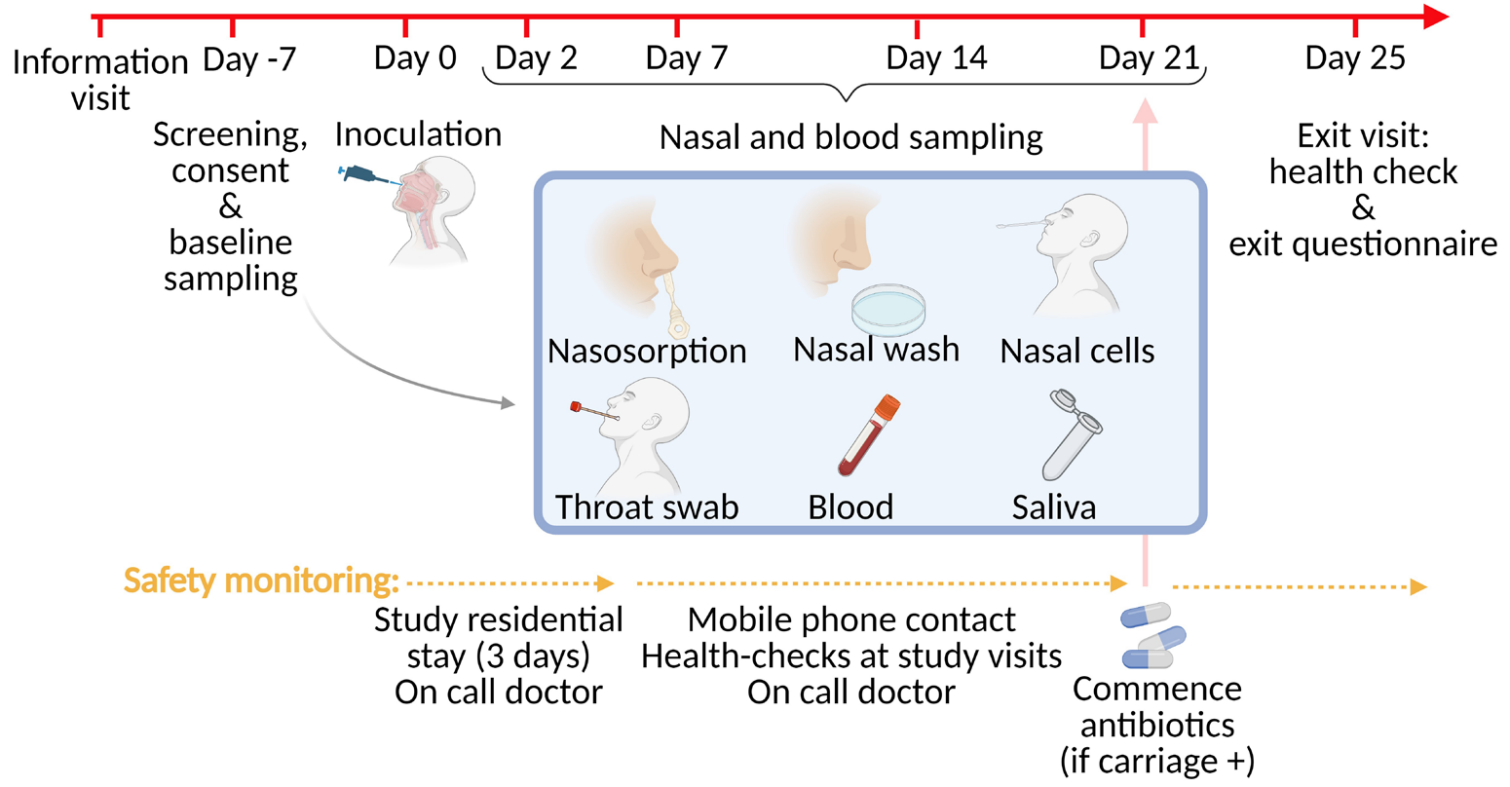
Study design, sample collection and safety monitoring at different time points. Participants complete an information visit after which they can contact the study team if they wish to participate. Screening, informed written consent, and baseline sampling occurs within 7 days of inoculation. Inoculation is following by follow up visits on days 2, 7, 14, and 21 for nasal and blood sampling. On day 25 participants undergo a final health-check and exit questionnaire based on a Likert-scale. Nasal and blood sampling includes nasosorption to collect nasal fluid, nasal cells collected using nasal curettage, a nasal wash, a throat swab, saliva, and blood for serum, and peripheral blood mononuclear cells. Safety monitoring post-inoculation includes a residential stay for three nights, health-checks at each study visit, mobile phone contact with study team, an on-call study doctor 24/7, and a safety pack which is distributed to each participant (thermometer, antibiotics, battery pack for mobile recharging, safety information leaflet).

This study will use the same experimental design well-established at the Malawi-Liverpool Wellcome programme and at Liverpool School of Tropical Medicine ^16, 17^. Successive cohorts will be inoculated in both nostrils with an escalating, controlled concentration of live, penicillin-sensitive, *Streptococcus pneumoniae* serotype 6B. Participants will be followed up for 21 days following inoculation during which sampling will occur at established time-points to establish pneumococcal carriage and cellular and humoral immune dynamics ( Figure 1). After 21 days, participants who exhibit pneumococcal carriage at any point during the study will commence a 3-day antibiotic course to clear the bacteria (participants may be advised by the clinical study team to commence antibiotics earlier if clinically indicated). A final health-check and exit interview will be conduction on day 25 to evaluate participant satisfaction with study participation.

### Primary outcomes

Study objective 1:

- Rate of solicited and unsolicited adverse events and serious adverse events recorded during scheduled and unscheduled study visits
- Participant satisfaction evaluated with a Likert-style questionnaire on day 25 post-inoculation

Study objective 2:

- Pneumococcal carriage density of serotype 6B on days 2, 7, 14, and 21 post-inoculation in PLHIV and HIV-negative controls

Study objective 3:

- Immunoglobulin G and A concentrations in nasal fluid or saliva respectively, and in blood plasma at baseline and on days 2, 7, 14, and 21 post-inoculation
- Exploratory longitudinal analysis of immune cell frequencies and cellular activation at nasal mucosa and peripheral blood at baseline and at days 2, 7, 14, and 21 post-inoculation

### Study setting

Study visits will be conducted in MLW clinical research rooms in Blantyre, Malawi. Study samples will be processed in the adjacent MLW laboratory.

### Participant recruitment

Seventy-five HIV-negative adults and 75 PLHIV aged 25 to 45 years old.

### Study duration

Recruitment commenced June 2023. Recruitment will be completed within 18 months.

### Participant schedule

**Figure 2.**
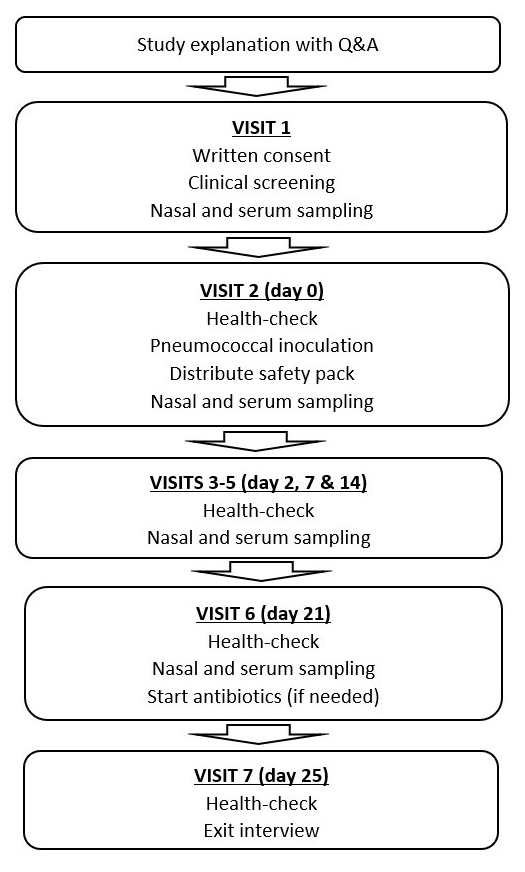
Flowchart of schedule study visits. Participants attend 7 study visits over 25 to 32 days. Prior to attending for their first study visit, all participants must complete an information visit in which participants are provided all necessary information regarding the study and participation. Participant understanding is evaluated using a quiz prior to obtaining informed consent. Study visit 1 (informed consent and clinical screening) occurs within 7 days of study visit 2 (inoculation). Nasal and blood sampling occurs at every study visit except study visit 7. Study visit 7 consists of a health-check and an exit interview on participants’ experience of participation.

Participants will attend twice prior to inoculation in order to establish fully informed and voluntary consent, and to evaluate participant eligibility and safety to participate ( Figure 2).

***Information visit:*** Interested candidates will attend for an information visit where they will receive a description of the study and its objectives, and an outline of participation and its associated risks and benefits. This information will be given to potential participants verbally and in written form in English and/or Chichewa. Candidates will then be allowed a ‘cooling off’ period to consider whether they want to participate. Those who still wish to participate are invited to contact the study team and will be scheduled for a screening visit.

***Screening and consent visit (study visit 1):*** Written consent will be obtained at this visit. A questionnaire will be conducted to ensure understanding of the study. Clinical screening will include a focused clinical history, a targeted clinical examination involving auscultation of the lung fields and heart sounds, blood sampling, a HIV antigen test if indicated, and a urinary pregnancy test if female. Blood samples will be obtained for full blood count, renal panel, and a HIV viral load and CD4 T cell count (if indicated) to evaluate participants’ fitness to participate in the study. Nasal cell and fluid samples and blood samples will be taken at this screening study visit to identify existing pneumococcal carriage, and to evaluate humoral and cellular immune markers. Participants naturally colonised with serotype 6B will not be eligible to continue in the study. Natural carriage with any other serotype can continue in the study. Should any previously unrecognised abnormality be identified during the screening visit, this will be explained to the individual, and appropriate investigations and follow-up will be arranged by the study team. Further participation will be determined at the discretion of the study doctor dependent on the nature of the abnormality detected. Eligible participants will be scheduled for their inoculation visit within 7 days of screening.

***Inoculation visit (study visit 2):*** Participants will be inoculated with a microbiologically and genetically confirmed antibiotic-susceptible *Streptococcus pneumoniae* serotype 6B strain BHN418. Inoculation will occur via a P200 micropipette containing 0.1 mL saline and a pre-specified dose of live pneumococcus. The inoculation dose will start at 20 000 colony-forming units per naris (cfu/naris). If a carriage rate of 50% is achieved in the first eight HIV-negative participants inoculated at this dose, all further participants will be inoculated at this dose. If the achieved carriage rate is below 50% then the dose will be escalated to 80 000 cfu/naris for the next eight HIV-negative participants. The dose may be further increased to 160 000 cfu/naris, then 320 000 cfu/naris, until the target carriage rate of 50% in HIV-negative participants is achieved. An adequate carriage rate is required in order to make statistical comparisons between participants, and in order to effectively evaluate vaccines in future studies using the model. Participants living with HIV will undergo the same dose escalation with close adverse event monitoring to establish safety in this population. The participant will be seated in a semi-recumbent position during inoculation. After inoculation, the participant will remain in this position for up to 15 mins and will be observed in the clinic room for 1 hour. Participants will receive a safety-pack including: i) A pocket-sized post-inoculation advice sheet with emergency contact details in English and Chichewa; ii) a digital thermometer and instruction on how to use it; iii) a course of amoxicillin with instructions of if and when to take them; and iv) a power-bank to ensure participants have access to a charged mobile phone to contact the study team if needed.

***Sampling visits (study visit 3-6):*** Participants will attend for sampling visits on days 2, 7, 14, and 21 post-inoculation. At each sampling visit a member of the study team will complete a structured study visit template electronically which includes a wellness-check, a review of symptoms, a review of vital signs, and a review of intervening antibiotic-use. Samples taken during these visits are detailed below.

***Exit visit (study visit 7):*** After the final sampling visit and completion of curative antibiotics (if indicated), participants will attend for an exit interview and final health check. The exit interview will be a structured electronic Likert-style questionnaire. The questions will cover five broad categories: Recruitment and consent; study procedures; study design and safety; study accommodation; and study participation.

If participants cannot attend on a particular post-inoculation study visit day then they can attend within 2 days of the scheduled visit.

## Methods

### Participant recruitment

Participants will be recruited from district health clinics in the local area. Potential participants will be signposted to phone or text a study team member (English and Chichewa-speaking) if they are interested in participating, and will be invited to an information meeting. Passive recruitment methods will allow participants to freely participate without feeling pressured by direct solicitation, as identified in the EHPC acceptability study ^18^.

### Inclusion criteria

- Aged 25 to 45 years old
- Fluent in spoken and written Chichewa or English
- Able to give informed written consent
- Access to a functional mobile phone
- Establish on antiretroviral therapy for ≥2 years (if PLHIV)
- Viral load below the lower limit of detection (<LDL) at screening (if PLHIV)
- CD4 count over 350 cells/mm³ at screening (if PLHIV)

### Exclusion criteria

- HIV-associated hospitalisation and/or treatment for major illness in preceding 2 years
- Currently under investigation for, or experiencing HIV-associated weight-loss, chronic diarrhoea, chronic cough, or another unexplained symptom
- Previous illness caused by pneumococcus
- Additional condition or medication impairing immune response or increasing risk of pneumococcal disease
- Living in close contact with an individual vulnerable to pneumococcal disease (e.g. child under 5 years old, adults aged over 65 years, pregnant woman)
- Allergy or intolerance to penicillin
- Acute illness in 7 days preceding inoculation
- Antibiotic course in last 2 weeks (excluding prophylactic antibiotics in PLHIV)
- Pregnant or trying to conceive
- Involved in another clinical study (unless observational or in follow-up)
- Current regular cigarette smoking (5+ cigarettes per week)
- Natural carrier of pneumococcus serotype 6B at screening visit
- Participants without a guardian

### Determination of colonisation

Pneumococcal colonisation will be assessed by conventional microbiological culture from nasal washes obtained from participants, and also confirmed by molecular methods (lytA polymerase-chain reaction-based methods). Pneumococcal colonisation is defined as the detection of pneumococcus serotype 6B by microbiological culture in nasal wash samples at any of the time points on days 2, 7, 14 and 21 post-inoculation, as previously described ^19^. The density of colonisation will be measured by classical microbiological culture and molecular methods.

### Sampling methods

Sampling methods used in this study have been successfully used during the PCV13 study at MLW, and are well-tolerated by participants ^2, 20^.

***Nasosorption:*** An absorptive strip is held inside the nose for 3 minutes to collect concentrated nasal lining fluid.

***Nasal wash:*** Five millilitres of sterile saline solution are instilled per naris with the participant in a semi-recumbent position. Participants are instructed to close their soft palate and hold the saline in the nares for a few seconds before leaning forward to allow the fluid to drip into a sterile Galli pot.

***Nasal curettage:*** The cup-shaped end of a plastic, single-use rhinoprobe is grazed along the lower nasal turbinate of each nostril to collect nasal cells.

***Throat swab:*** Simple posterior pharyngeal swabbing on a dry swab for viral co-infection testing.

***Venepuncture:*** Venepuncture will be performed to ensure participants are fit to participate, to evaluate peri-inoculation serum levels of commonly used antibiotics, and to evaluate peripheral blood immune dynamics.

***Saliva:*** salivette held for saturation or a spit in the tube.

### Safety monitoring

This will be the first CHIM study in PLHIV and participant safety is paramount. Experimental pneumococcal carriage is expected to be a benign event due to the pervasiveness of natural pneumococcal carriage in PLHIV and the low carriage to disease ratio. In the unlikely event that invasive pneumococcal disease develops in a study participant, robust safety monitoring will identify signs of invasive disease early and enable immediate treatment. Participants will be housed in study accommodation for the first three days following inoculation, as invasive disease tends to occurs early during carriage acquisition. Participants will have a symptoms-review and vital-signs check at every study visit. Any abnormalities will trigger a formal clinical review by a study doctor. In between study visits, participants will have phone contact with the study team to establish that they are well and apyrexial. An escalation protocol is established if participants are not contactable. An on-call doctor is available for participants 24/7 and participants are given written and verbal instruction on when and how to contact the doctor. All participants will be required to have a mobile phone, and will be issued a power-bank to keep their phone charged during power-outages. Participants will be issued an antibiotic course at the point of experimental inoculation and will be instructed to keep them on their person, and on when/if to take them. The pneumococcal isolate used during experimental inoculation is microbiologically and genetically confirmed antibiotic-susceptible. Participants will have the relevant safety information written in English and Chichewa on laminated, pocket-sized cards.

### Termination of carriage

Participant exhibiting pneumococcal carriage at any point during the study will be advised to commence a 3-day course of amoxicillin at study-end.

### Immunological measurements

Surface antigen staining of nasal cells will occur in real-time within 4 hours of collection to characterise the major nasal cell populations and their frequency at different time points of the study. Nasal fluid will be stored and will be analysed in batches using a commercial multiplex cytokine assay. Serotype-specific immunoglobulin G and A concentrations will be evaluated in blood, nasal wash and saliva respectively using antibody enzyme-linked immunosorbent assay (ELISA). Bulk RNA analysis will be performed on a subset of nasal samples, and single-cell sequencing will be performed on a small number of nasal cells collected using nasal swabs. Similar immune evaluations will be performed on blood to determine whether immune processes are local to the nasal mucosa, or from systemic spill-over.

### Sample size calculation

The study will recruit 75 PLHIV and 75 HIV-negative controls aged 25–45 years old. The sample size is based on 50% carriage rate following inoculation in HIV-negative controls ^17^, and a 50% greater carriage rate amongst PLHIV based on community carriage point-prevalence ^21^. 132 participants (66 per group) will be required to achieve 80% power with p=0.05 so 150 participants will allow for some participant drop-out.

### Statistical analysis

Descriptive statistics will be used to describe group characteristics and to describes rate of pneumococcal carriage in both groups. Pneumococcal carriage rate will be described by area-under the curve of carriage density over the duration of follow up, and as a binary carriage positive (at any time) compared to carriage negative. Natural carriage and baseline serum concentrations of commonly used antibiotics (e.g. cotrimoxazole) will be included as a variable in the regression. Two-tailed comparisons will be performed to evaluate density and activation of immune cell populations, and immunoglobulin concentrations between those who become colonised with pneumococcus and those who do not in PLHIV and HIV-negative controls. Paired comparisons will be performed to compare density and activation of immune cell populations before and after pneumococcal inoculation. Multiple comparisons will be adjusted for using Benjamini-Hochberg or Bonferroni methods ^22^.

### Adverse event and serious adverse event monitoring

Reported and solicited adverse events (AEs) will be collected systematically during the research and recorded in the case report form. Any serious adverse event (SAE) occurring to a research participant will be reported within 24 hours to an independent Data, Safety and Monitoring Board (DSMB), to the ethical review board, and to the study sponsor. The DSMB consists of three professors of infectious diseases, and one professor of statistics based in Malawi and internationally. In the event of an SAE (death, hospitalisation, life-threatening, and significant disability), the research will be stopped temporarily for investigation and any further work deferred until advice has been provided.

## Ethical approval

The study protocol has been approved by the National Health Sciences Research Committee of Malawi (22/09/3053), and by the Liverpool School of Tropical Medicine Research Ethics Committee (22-077).

## Study sponsorship and funding

The study is sponsored by Liverpool School of Tropical Medicine. It is funded by the Wellcome Trust (grant numbers 226731/Z/22/Z and 211433/Z/18/Z).

## Study registration

Clinicaltrials.gov registry number NCT05698225.

## Remuneration

Participants will be compensated for time, transport, subsistence, and discomfort experienced. Remuneration amounts have been calculated using local Malawi College of Medicine Research Ethics Committee guidelines ^23^. Participants will not be remunerated for the information visit. Remuneration will be given in cash at each study visit (18 500 Malawian Kwacha per visit, 130 000 Malawian Kwacha if all study-visits are completed).

## Confidentially and anonymity

Only authorised members of the research team will have access to any personal information. Only information of direct relevance to the study will be collected. All electronic records containing personal information will be stored in a password protected database on a password protected server at MLW. Electronic case report form data will be collected on encrypted study-specific tablet devices and synchronised daily onto the MLW server. Each researcher will only be able to view the information required to fulfil their role in the study. This approach is well established at MLW, and the policy governing MLW data management policy is available upon request. Paper documentation containing personal information will be kept in a locked filing cabinet in a locked room in the Queen Elizabeth Central Hospital research clinic. Each participant will be assigned a unique non-identifiable study number by a member of the clinical research team at recruitment. Unlinked non-identifiable clinical data will be stored and analysed at the MLW-laboratories and collaborating laboratories.

## Samples and data

MLW will act as custodian for all data and samples collected during the study. Consent will be obtained from the participant to use the samples for this research only. Samples will be stored for a maximum of five years. Laboratory samples will also be sent to national and international collaborating laboratories to utilise specialist expertise not available in Malawi. Samples will be labelled with the anonymised study number. Consent will specifically be obtained from the participants to allow samples to be sent to our collaborators.

## Dissemination of findings

The findings from this study will be disseminated amongst the scientific community. We intend to publish our findings in peer-reviewed scientific journals and present data at appropriate local, national and international conferences. We will produce a close-out report for the ethical review committees at the end of the study and a final report once data are published. In addition, we will produce a lay report of our findings which will be made available to all participants.

## Discussion

PLHIV remain a key population to consider when addressing the burden of pneumococcal disease, particularly in settings with high HIV prevalence. They are at risk of disease themselves and are at risk of driving onward transmission of the bacteria in vaccinated communities. An evidence-base to guide vaccination policy is lacking. A trial of Ugandan adults with PLHIV demonstrated increased rates of pneumococcal disease associated with PPV23 vaccination ^12, 13^. A trial of Malawian adults with PLHIV demonstrated safety and efficacy against vaccine-serotype disease with PCV7 administation ^14^. However, PCV7 has limited serotype coverage and gives rise to ‘serotype replacement’ in which an increase in non-vaccine serotype disease limits overall vaccine effectiveness against pneumococcal disease ^15^. The British HIV Association and European AIDS Clinical Society recommend the routine use of PCV13 in PLHIV but do not recommend against giving PPV23 if administration meets other national recommendations (e.g. vaccinating over 65 year olds) ^24, 25^. The National Institute of Health recommends PCV15 with PPV23 at least 8 weeks later, or PCV20 ^26^. Guidelines for a sub-Saharan African settings are absent. This study will transform the evidence-base for pneumococcal vaccination in PLHIV in sub-Saharan Africa by establishing a model for efficient vaccine-efficacy trials, and by identifying the immune correlates of protection from pneumococcal carriage to guide more effective vaccine immunogenicity evaluations.

This will be the first CHIM study in PLHIV and participant safety is paramount. Natural pneumococcal carriage does not often result in invasive disease, and is a ubiquitous event in the study population. The point prevalence of natural pneumococcal carriage in PLHIV in Malawi ranges between 26% and 52%, depending on urban or rural settings ^5, 6, 9, 27^. In a six to ten month period, 99% of PLHIV will experience at least one pneumococcal carriage event, and on average adults will experience five separate pneumococcal carriage events ^28^. Meanwhile, the rate of pneumococcal disease in sub-Saharan Africa in the post-ART era is estimated at 318 per 100 000 population ^29^. Pneumococcal carriage increases with the initiation of ART, compared to the ART-naïve, meaning as ART is rolled-out across sub-Saharan Africa, pneumococcal carriage rates are increasing ^5, 6^. Meanwhile, pneumococcal disease rate is reduced by a half to a third by ART, and rates of HIV-associated hospital admissions in Malawi have halved as ART is rolled out in the country ^30, 31^. Thus it can be inferred that the ratio of natural pneumococcal carriage to pneumococcal disease is very small and improves on ART. Experimental carriage is expected to be a similar benign event. Safety in this study is further enhanced by careful participant selection and by pneumococcal serotype selection. The exclusion criteria ensure that participants are well and stable on antiretroviral therapy. Risk of developing pneumococcal disease is strongly associated with CD4 T cell count ^32^, and all participants in this study will be immune-reconstituted and without additional risk factors for invasive pneumococcal disease. The pneumococcal serotype 6B used in the study has been selected for a lower carriage to disease ratio than other serotypes ^33^. Less than 2% of invasive pneumococcal isolates in Malawi are caused by serotype 6B and are largely seen in children if at all ^34, 35^.

Public and participant perception are a key consideration for CHIM studies in sub-Saharan Africa. The acceptability of the EHPC model to Malawian medical, research, and community stakeholders has been evaluated and provides a framework for this study to address ethical and safety considerations ^18^. For example, the study will use passive recruitment tactics to avoid potential participants feeling pressured by direct solicitation, as identified by stakeholder consultation. Reasons for participation in EHPC studies have been explored in detail in Malawi and provides guidance for avoiding undue solicitation during recruitment ^36^. Conducting a CHIM in PLHIV has been positively perceived by HIV stakeholders in Malawi during preliminary evaluations. The importance of giving PLHIV the opportunity to participate in research for the sake of equity and autonomy has been highlighted, while the need to balance risk to participants is recognised. Acceptability will be explored in a stand-alone study using qualitative methods running concurrently with this study.

## Conclusion

This first CHIM in PLHIV can be conducted safely and is acceptable to local stakeholders. The findings from this study will explain high pneumococcal carriage rates observed in PLHIV and will identify potential strategies to address the issue. The study will facilitate effective and efficient pneumococcal vaccine evaluations in PLHIV, and thus guide policy makers in addressing the burden of pneumococcal carriage in PLHIV in sub-Sharan Africa.

## Data Availability

No data are associated with this article.
