# Supplementary material for: Experimental pneumococcal carriage in people living with HIV in Malawi: the first controlled human infection model in a key at-risk population
Source: Wellcome Open Res. 2024 Jan 3;9:2. [Version 1] doi: 10.12688/wellcomeopenres.19949.1 (PMC10864820; doi:10.12688/wellcomeopenres.19949.1)
Supplement: Peer review history

# Reviewer report: Reviewer response for version 1

**DOI:** 10.21956/wellcomeopenres.22093.r72499

**Published:** 2024-02-23

**Authors:** Michaela Tinggaard

**Affiliations:** 1 Infectious Diseases, Copenhagen University Hospital, Rigshospitalet, Copenhagen, Denmark

This paper describes the clinical study protocol for an EHPC model used in healthy adults with and without HIV living in Malawi. The participants will be inoculated intranasally with S. pneumonia type 6B with increased doses with the objectives of establishing an optimal safe dose; measure carriage density and to identify immune correlates of protection up to 21 days post-inoculation.  The study is of high relevance as it is the first study to examine experimental pneumococcal carriage in PLHIV - a population of high risk of invasive pneumococcal disease, which have been shown to exhibit sub-optimal responses to pneumococcal immunization. 

 I have minor comments.

Introduction:

 - "(..) for a disease that continues to (..)" - maybe specify earlier on that the disease in question is pneumococcal disease. 

 - "26 to 52% of PLHIV in sub-Saharan Africa"; could be compared to carriage rates in people without HIV

 -  "such as pneumonia" - pneumonia do not classify as IPD. 

 - "associated with harm i PLHIV" - would change to 'possibly associated to'

 - Regarding PCV7; this argument seems a little outdated with PCV13 / PCV20 being available

 - Is reference 15 correct? 

Study protocol:

 - If participants are colonized with other serotypes than 6B at screening, they are still included. Could this affect results and how? Could be addressed in the discussion.

 - Exclusion criteria: Do you consider vaccination status?

 - "invasive disease tends to occurs early during carriage acquisition" - reference?

 - Termination of carriage: Why a 3 day course? 

Discussion

 - "Guidelines for sub-Saharan African settings are absent". It could be addressed why policies from other settings could/should not be implemented in SSA?

 - Would modify statements regarding carriage and initiation of ART as evidence (few studies) suggest this association. 

Conclusion:

 Would again modify: "The findings will explain (..) " to i.e. 'the findings will contribute to (..)'.

Is the study design appropriate for the research question?

Yes

Is the rationale for, and objectives of, the study clearly described?

Yes

Are sufficient details of the methods provided to allow replication by others?

Yes

Are the datasets clearly presented in a useable and accessible format?

Not applicable

Reviewer Expertise:

Pneumococcal carriage studies among adults and immunogenicity following pneumococcal vaccination in PLHIV.

I confirm that I have read this submission and believe that I have an appropriate level of expertise to confirm that it is of an acceptable scientific standard.

# Reviewer report: Reviewer response for version 1

**DOI:** 10.21956/wellcomeopenres.22093.r72495

**Published:** 2024-02-13

**Authors:** Robert KM Choy

**Affiliations:** 1 Center for Vaccine Innovation and Access, PATH, Washington, USA

This manuscript reports the clinical study protocol for the first controlled human infection model (CHIM) of S. pneumoniae that is being conducted in HIV-infected subjects in Malawi. The authors have previously reported use of this model to infect healthy individuals and are now extending this to an at-risk population. Because this model is expected to only result in carriage of pneumococcus, rather than symptomatic disease as with some other CHIMs, this study is considered ethical to conduct in this population.

 The manuscript is generally clear and well-written. I have a couple suggestions that may improve the clarity:

 1. In the Study Protocol section under Primary outcomes, there are five study objectives listed. However, it doesn't seem to me that these are all in fact Primary outcomes of the study, for example the fifth objective is described as Exploratory. I suggest that the authors clarify which outcomes are Primary, Secondary, and Exploratory. Furthermore, these should be clearly referred to as outcomes, rather than objectives, particularly because there is an earlier subsection titled "Study objectives".

 2. The dose-escalation plan from 20,000 to 320,000 cfu/naris is included in the description of the Inoculation visit (study visit 2). I suggest (either here or in the Discussion) a recap of the attack rates previously observed in other studies with the proposed doses of this strain of pneumococcus and what, if any, differences are expected in this study, either in HIV infected or uninfected subjects.

Is the study design appropriate for the research question?

Yes

Is the rationale for, and objectives of, the study clearly described?

Partly

Are sufficient details of the methods provided to allow replication by others?

Yes

Are the datasets clearly presented in a useable and accessible format?

Not applicable

Reviewer Expertise:

My areas of research include vaccine development and the development of controlled human infection models for infectious diseases of global health importance.

I confirm that I have read this submission and believe that I have an appropriate level of expertise to confirm that it is of an acceptable scientific standard.

# Reviewer report: Reviewer response for version 1

**DOI:** 10.21956/wellcomeopenres.22093.r72497

**Published:** 2024-02-13

**Authors:** Sophie Belman

**Affiliations:** 1 Global Health Resilience; Earth Sciences Department, Barcelona Supercomputing Center (Ringgold ID: 132144), Barcelona, Catalonia, Spain; 2 Parasites and Microbes, Wellcome Sanger Institute (Ringgold ID: 47665), Hinxton, England, UK

This paper describes a protocol for a pneumococcal challenge model in people living with HIV (PLHIV) in sub-Saharan Africa. This protocol will allow proper evaluation of pneumococcal vaccines and immune correlates for protection in PLHIV. The protocol compares pneumococcal acquisition among people with and without HIV and entails challenge with serotype 6B (known to be rarely responsible for pneumococcal disease). This study includes housing and monitoring for the first several days after infection (the time when disease is most probable), regular monitoring following that up to 21 days. Nasal fluid, mucosal, and blood samples will be taken on days 2, 7, 14, and 21 days. The study objectives are clearly outlined and include 1) establishing the safety of such a challenge model in PLHIV, 2) comparing the rate and density of acquisition in PLHIV and people without HIV, and 3) determining the immune correlates of protection in PLHIV. This study includes continuous monitoring throughout the period highlighted by the provision of power banks to participants to ensure the ability to contact the study team given lack of electricity. It is a well thought through protocol with appropriate attention both to ethics of recruitment as well as appropriate monitoring of study participants. It will reveal important information about how PLHIV responds to vaccines and appropriate measurements for that response. The authorship team are experienced with human challenge models and it is exciting to see a protocol which might make a huge difference in ability to mitigate disease in this at-risk population.

 I have a few specific comments and questions. I have written the header below which I am referring to. Sections which I have not referred to explicitly are sections for which I have no comments or questions. 

Abstract: Where the authors have written “Nasal mucosal” it should say “Nasal mucosa”

Introduction: Only PPV23 and PCV7 are addressed in the introduction. Are there other studies of additional serotype coverage in PLHIV? Addressing the use of other vaccines (PCV15, PCV20) explicitly would be appropriate even if just to say their impact is unknown. Some of what is in the discussion with regards to vaccine use in PLHIV would be helpful in the introduction instead. 

Study Design: Is a three day course enough to completely clear infection, is there an appropriate citation for this? The antibiotic course begins on day 21 if carriage positive, is there sampling again on day 25 to determine if carriage is cleared. If carriage is not cleared will follow-up sampling continue? Please be explicit here.

Participant Recruitment: Of 75 participants how many are likely to become carriers? Is this enough to allow appropriate statistical analyses? Please justify these numbers.

Inoculation Visit: I presume a 50% rate of carriage in 8 participants implies 4/8 participants will be carrying. Please confirm that this is what you mean and make it more explicit in the text. This may be appropriate to confirm later in the “Sample Size Calculation” section.

Exclusion Criteria:  Participants without a guardian”. If all participants are between 25 and 40 what is meant by guardian? I do not expect independent adults between these ages to have a guardian.

Safety Monitoring: The second sentence in this paragraph requires a citation as is found in the discussion. Again, some of what is in the discussion with regards to point prevalence in PLHIV should be moved to the introduction due to your reference of it here. Where it says a “symptoms-review” it should say “symptom-review”

Termination of Carriage: While serotype 6B is typically benign will there be additional follow-up to determine that clearance with antibiotics was successful in this study?

Discussion: Some of what is in the discussion would be more useful in the introduction. Predominantly the reference to the high rates of carriage in PLHIV, the increasing carriage rates with the use of ART, the recommendations for PCVs beyond PCV7 in PLHIV, and the low virulence of serotype 6B. It would be helpful to understand how the protocol is designed to have read those in the introduction.

 Additional queries I have regard whether the carriage density will be monitored for serotypes other than 6B if participants are already carrying. This may allow some understanding of the interaction between different serotypes in co-carriage in this population.

Is the study design appropriate for the research question?

Yes

Is the rationale for, and objectives of, the study clearly described?

Yes

Are sufficient details of the methods provided to allow replication by others?

Yes

Are the datasets clearly presented in a useable and accessible format?

Not applicable

Reviewer Expertise:

Pneumococcal evolution/fitness dynamics, genomics, mathematical modelling, transmission and migration dynamics of the pneumococcus

I confirm that I have read this submission and believe that I have an appropriate level of expertise to confirm that it is of an acceptable scientific standard.
